# Differences in kinetic factors affecting gait speed between lesion sides in patients with stroke

**DOI:** 10.3389/fbioe.2024.1240339

**Published:** 2024-03-19

**Authors:** Yusuke Sekiguchi, Dai Owaki, Keita Honda, Shin-Ichi Izumi, Satoru Ebihara

**Affiliations:** ^1^ Department of Physical Medicine and Rehabilitation, Graduate School of Medicine, Tohoku University, Sendai, Japan; ^2^ Department of Robotics, Graduate School of Engineering, Tohoku University, Sendai, Japan; ^3^ Department of Physical Medicine and Rehabilitation, Graduate School of Biomedical Engineering, Tohoku University, Sendai, Japan; ^4^ Department of Internal Medicine & Rehabilitation Science, Disability Sciences, Tohoku University Graduate School of Medicine, Sendai, Japan

**Keywords:** stroke, gait, kinetics, lesion side, laterality, kinetic coordination

## Abstract

The differences in kinetic mechanisms of decreased gait speed across brain lesion sides have not been elucidated, including the arrangement of motor modules reflected by kinetic interjoint coordination. The purpose of this study was to elucidate the differences in the kinetic factors of slow gait speed in patients with stroke on the lesion sides. A three-dimensional motion analysis system was employed to assess joint moment in the lower limb and representative gait parameters in 32 patients with right hemisphere brain damage (RHD) and 38 patients with left hemisphere brain damage (LHD) following stroke as well as 20 healthy controls. Motor module composition and timing were determined using principal component analysis based on the three joint moments in the lower limb in the stance phase, which were the variances accounted for principal components (PCs) and the peak timing in the time series of PCs. A stepwise multiple linear regression analysis was performed to identify the most significant joint moment and PC-associated parameter in explaining gait speed. A negligible difference was observed in age, weight, height, and gait speed among patients with RHD and LHD and controls. The following factors contributed to gait speed: in patients with RHD, larger ankle plantarflexion moment on the paretic (*p* = 0.001) and nonparetic (*p* = 0.002) sides and ankle dorsiflexion moment on the nonparetic side (*p* = 0.004); in patients with LHD, larger ankle plantarflexion moment (*p* < 0.001) and delayed peak timing of the first PC (*p* = 0.012) on the paretic side as well as ankle dorsiflexion moment on the nonparetic side (*p* < 0.001); in the controls, delayed peak timing of the first PC (*p* = 0.002) on the right side and larger ankle dorsiflexion moment (*p* = 0.001) as well as larger hip flexion moment on the left side (*p* = 0.023). The findings suggest that the kinetic mechanisms of gait speed may differ among patients with RHD following patients with stroke with LHD, and controls.

## 1 Introduction

Patients with stroke have reduced gait speed, which impairs their mobility within the community (Fulk et al., 2017), limits their living space (Tashiro et al., 2019), compromises their independence in daily life (Compagnat et al., 2021), and hinders their ability to resume to work (Jarvis et al., 2019). This can ultimately affect their overall quality of life (Sprigg et al., 2013). Gait speed is a crucial indicator of functional mobility and is associated with various aspects of daily life for patients with stroke.

Current interventions, such as electromechanical-assisted gait training and treadmill training with physiotherapy, have shown a modest increase in walking velocity for patients with stroke (Mehrholz et al., 2017; Mehrholz et al., 2020). However, these improvements may not reach the minimal clinically significant changes for gait speed, which range from 0.10 to 0.18 m/s (Fulk et al., 2011; Bohannon and Glenney, 2014). This suggests that current interventions may be insufficient. Various factors, such as ankle moment and trail limb angle, contribute to reduced propulsion force in patients with stroke (Hsiao et al., 2016b), indicating different mechanisms at play when it comes to increasing gait speed. Trail limb angle is defined as the angle between the laboratory’s vertical axis and the vector connecting the greater trochanter and the fifth metatarsal head. Therefore, understanding individual-specific factors contributing to reduced gait speed is crucial for developing personalized training strategies.

Various studies have examined the relationship between gait speed and the side of the brain lesion in patients with stroke, but findings have been inconsistent (Chen et al., 2014; Kim et al., 2019; Ursin et al., 2019; Frenkel-Toledo et al., 2021; Vismara et al., 2022). Some studies found that patients with right hemisphere brain damage (RHD) have slower gait speed than those with left hemisphere brain damage (LHD), while others found no significant difference. Patients with RHD often exhibit decreased capacity to shift body weight and unstable body movement and posture control, which can lead to slower start and reduced muscle activation in the paretic leg (Fernandes et al., 2018; Coelho et al., 2019). These patients also show higher center of pressure (CoP) sway velocity during static standing (Fernandes et al., 2018). Hsiao et al. (2017) suggested that difficulties in transferring weight from side to side and maintaining stability while walking could decrease walking speed (Hsiao et al., 2017). This process leads to the generation of vertical ground reaction force, resulting in an increase in walking speed and control of whole body angular momentum (WBAM) in the frontal plane during gait (Silverman and Neptune, 2011; Hsiao et al., 2017). Patient with strokes often exhibit increased WBAM during their gait (Nott et al., 2014; Brough et al., 2019). The ankle plantar flexion moment in late stance, which begins when the foot contacts the ground and ends when the foot leaves the floor, was related to the vertical ground reaction force and WBAM during gait (Silverman and Neptune, 2011; Elhafez et al., 2019). These observations suggest that the reduction in walking speed and increase in WBAM observed in patients with RHD may be due to a significant decrease in ankle plantar flexion moment in late stance on the paretic side during gait. However, this is only a tentative explanation and further research is needed to confirm or refute this hypothesis.

Previous researches have shown that in patient with strokes, walking speed is linked to kinetic parameters in the paretic lower limb, particularly at the ankle and hip joints (Olney et al., 1994; Kim and Eng, 2004; Jonkers et al., 2009; Sekiguchi et al., 2012; Mentiplay et al., 2019). From the results of our previous study using principal component analysis (PCA) in healthy controls, we found that as gait speed increases, the later peak timing of the first principal component (PC) demonstrates that the timing of propulsion control, exhibited by kinetic coordination, plays an important role in generating propulsion (Sekiguchi et al., 2019). In patients with stroke, we have observed a decrease in ankle joint moment and disrupted kinetic coordination, which impacts their forward movement, using PCA (Sekiguchi et al., 2022). Additionally, the first PC during gait, which involves moments at the ankle and hip joints, occurs earlier in time and includes knee joint flexion or extension (Sekiguchi et al., 2022). However, it remains unclear whether there is a relationship between the timing of the first PC and gait speed in patients with stroke.

The purpose of this study was to elucidate the differences in the kinetic factors of slow gait speed between the lesion sides in patients with stroke. We conducted a stepwise multiple linear regression analysis to determine which joint moment and parameter associated with kinetic coordination are most explanatory for gait speed. In patients with right hemisphere damage (RHD), we hypothesize that the observed reduction in walking speed and increase in whole body angular momentum (WBAM) may be due to a significant decrease in the ankle plantar flexion moment in the late stance on the paretic (left) side during gait. Furthermore, in patients with left hemiplegia, difficulties in dynamic control may result in a lack of correlation between walking speed and kinetic coordination on the paretic (right) side.

This study provides valuable insights into the kinetic mechanisms underlying decreased gait speed in patients with stroke and may inform future rehabilitation strategies. By identifying specific joint moments and parameters associated with decreased gait speed, rehabilitation professionals may be able to develop more targeted interventions to improve walking speed and functional mobility in patients with stroke.

## 2 Materials and methods

### 2.1 Subject

The present study included 32 patients with right-sided (8 females, 58 ± 10 years old, Table 1) and 38 patients with left-sided (9 females, 54 ± 13 years old, Table 1) brain lesions following stroke as well as 20 healthy controls (8 females, 57 ± 16 years old, Table 1). All patients underwent post-stroke rehabilitation, which was tailored to each patient’s needs and recovery phase. To be included, patient with strokes had to meet the following criteria. (1) being able to walk without a cane over a distance of at least 7 m, (2) experiencing paresis ranging from mild to severe, with a Brunnstrom recovery stage of VI or lower in the lower limb on the paretic side, and (3) having an ischemic or hemorrhagic supratentorial lesion. To be included as a control, healthy controls must not have had any neurological lesions. Healthy controls were not eligible if they had any of the following: (1) medical conditions that were not stable, (2) a history of major orthopedic surgery or current orthopedic conditions that could affect their ability to walk, or (3) higher brain dysfunction that could affect the accuracy of the measurements. Before participating in this study, the participants gave their written and informed consent. Our institutional review board approved this study (2016-1-354).

**TABLE 1:** Subjects’ demographic characteristics.

	Right-sided brain lesion	Left-sided brain lesion	Controls
N	32	38	20
Gender (Male/Female)	24/8	29/9	12/8
Age (years)	58.6 ± 9.9	53.9 ± 13.0	57.4 ± 16.3
Height (cm)	165.3 ± 9.0	165.2 ± 7.5	165.9 ± 8.7
Weight (kg)	62.3 ± 9.1	64.8 ± 11.4	61.6 ± 11.1
Diagnosis (Hemorrhage/Infarction)	22/10	21/17	
Location of lesion (M/S)	3/29^d^	8/29	
Time since stroke (months)	26.8 ± 39.3	31.4 ± 40.5	

^a^ Values are expressed as means ± standard deviations.

^b^ No significant difference was observed in the physical characteristics, diagnosis, location of lesion and time since stroke among the groups.

^c^ M/C represents mixed cortical & subcortical and cortical lesions.

^d^ One patient with left hemisphere damage did not have CT or MRI images available as they were hospitalized in another facility.

### 2.2 Gait analysis

**FIGURE 1:**
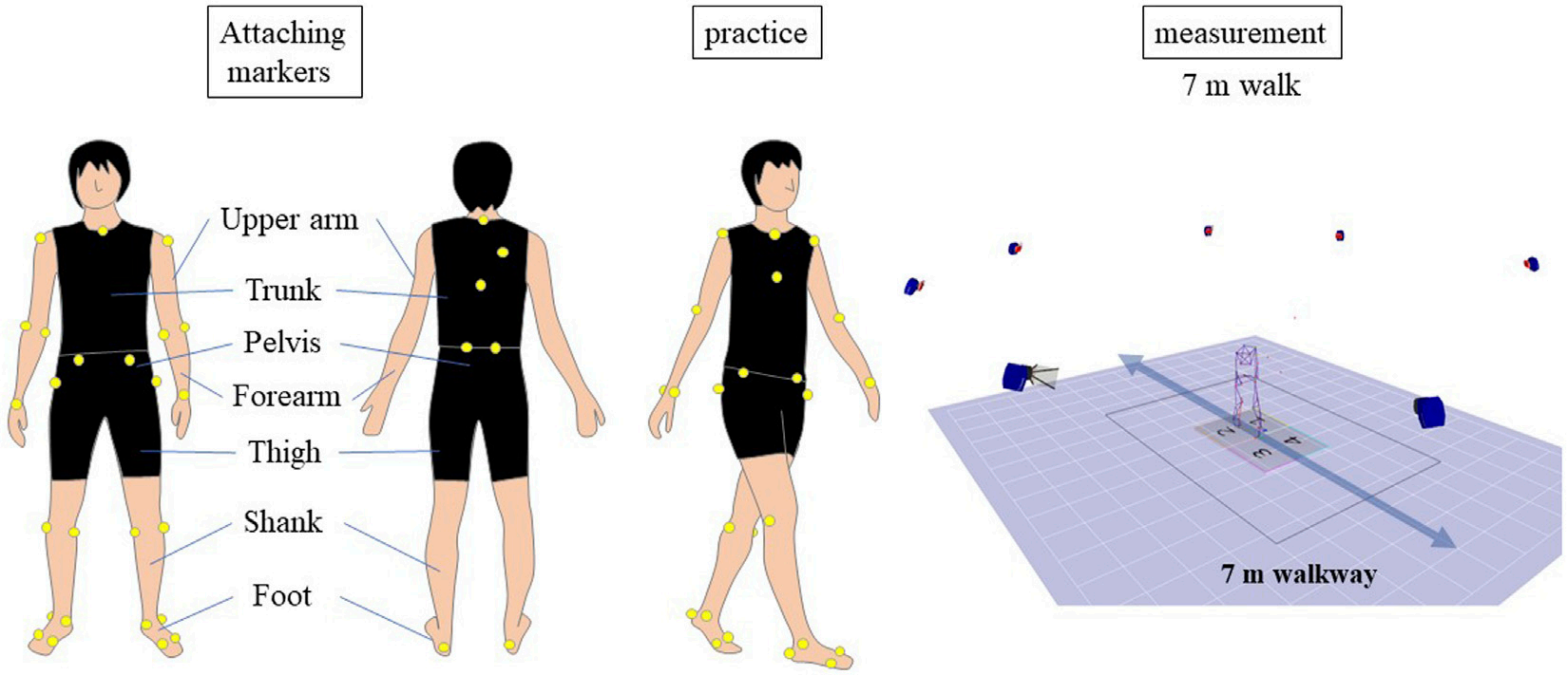
Gait Analysis Procedure. The diagram illustrates the experimental setup for the gait analysis study. Participants walked a 7-meter distance multiple times, with data from an average of more than five strides used for analysis. Reflective markers were placed on 12 body segments, and motion data were collected using an 8-camera motion analysis system. Ground reaction force data were collected using four force plates embedded in the walkway.

The participants were asked to walk 7 m, repeating the task 2 to 10 times until data for five strides were collected. The patients walked barefoot at a comfortable pace without assistive devices and could rest between trials if needed. The walking speed of the healthy subjects over a distance of 7 m was determined using the patient’s previously recorded walking speed as a reference, and they were instructed to walk the distance within that time. Participants had the opportunity to practice walking before the measurement (Figure 1). We calculated the duration required for the healthy individuals to traverse a distance of 7 m, aligning it with their previously recorded walking pace. The healthy subjects were guided to cover the 7-meter distance within the predetermined time frame. Prior to the actual gait measurement, the healthy controls rehearsed the 7-meter walk multiple times. Data from an average of more than five strides from successful trials were used for analysis. Whole-body motion data were collected using an 8-camera motion analysis system at a rate of 120 Hz (MAC 3D, Motion Analysis Corporation, Santa Rosa, CA, USA) with 33 reflective markers placed on 12 body segments (As shown in Supplementary Table). The three-dimensional coordinates were smoothed with a bidirectional fourth-order Butterworth low-pass filter with a cutoff frequency of 6 Hz. Ground reaction force data were collected at a rate of 1,200 Hz using four force plates (Anima Corporation, Chofu, Tokyo, Japan) embedded in the walkway and smoothed with a bidirectional fourth-order Butterworth low-pass filter with a cutoff frequency of 200 Hz.

A model consisting of 12 body segments, based on anthropometric data and following the work of (Dumas et al., 2007), included the feet, shanks, thighs, pelvis, thorax, upper arms, and forearms. A joint coordinate system was used to calculate the kinematic data for each joint in the lower extremities, as described by Winter (2009).

Additionally, inverse dynamics was employed to estimate the kinetics of the joints in the lower extremities (Selbie et al., 2013). All kinematic and kinetic data were normalized to 100% of a single gait cycle. The representative method was used to calculate spatiotemporal parameters (Butler et al., 2006). The kinematic and kinetic data were used to obtain the representative gait parameters, following the methods outlined in a previous study by Kinsella and Moran (2008). The kinetic data was normalized to the patient’s body weight.

A spatiotemporal decomposition using principal component analysis (PCA) was performed on the joint moments in the lower limb (ankle, knee, and hip) to calculate the coordination of the lower limb joints and the loading on each joint. This is the same method used in our previous study (Sekiguchi et al., 2022). The kinetic data in the stance phase during gait was used in this study unlike one gait cycle used in the previous study (Sekiguchi et al., 2022). The parameters of interest included the percentage of variance explained by each principal component (PC), the timing of the peak of the first PC, and the factor loadings of each joint in each PC. The percentage of variance explained by each PC and the peak timings represented the spatial and temporal aspects of the motor module.

The evaluation of balance control was conducted using the range of whole-body angular momentum (WBAM_R_) in the frontal plane (Brough et al., 2019). The calculation of whole-body angular momentum (WBAM) was performed using a 12-segment inverse dynamics model. This involved aggregating the angular momentum of each body segment around the center of mass for the entire body in the frontal plane. The whole-body angular momentum was then normalized based on the subject’s mass, walking speed, and leg length. WBAM_R_ was characterized as the difference between the maximum positive and minimum negative peaks of WBAM, with an average taken across all strides.

All gait-related parameters were calculated using a custom software program created with MATLAB (MathWorks Inc., Natick, MA, USA).

### 2.3 Clinical characteristics

A physical therapist, Y.S., assessed the neurological impairment of patients using the Stroke Impairment Assessment Set (SIAS) (Tsuji et al., 2000). Information about the patients’ demographic and clinical characteristics was gathered through interviews and medical records.

### 2.4 Statistical analysis

The number of gait cycles used for statistical analysis varied from 5 to 9 for each participant. The determination of the number of gait cycles was based on each patient’s walking ability. In instances where a slower walking speed and high variability were expected, we incorporated a larger number of steps into our analysis. Gait speed, cycle time, stride length, step width, and WBAM_R_ were compared between the three groups (RHD and LHD patients and controls) using one-way analysis of variance (ANOVA). Stance, swing, double stance, single-support phase times, step length, joint angle, joint moment, and PCA-related parameters were analyzed using a two-way ANOVA. Although we evaluated the normality of the gait dataset using the Shapiro-Wilk test, we did not conduct non-parametric tests for the two-way ANOVA, as these tests do not compute interactions between factors. The within-subject factor was side (paretic/nonparetic for hemiparesis patients and right/left for controls) and the independent factor was group (hemiparesis patients/controls). The two-way ANOVA was performed separately for left-sided and right-sided brain lesions. If a significant difference was found, a Bonferroni *post hoc* test was conducted. In addition, we compared all parameters between patients with RHD and LHD using unpaired t-test. A chi-square test of independence was conducted to investigate the relationship between the side of the lesion and the location of the lesion and the diagnosis. Stepwise multiple linear regression was used to determine which joint moments and PCA-related parameters best explained gait speed. Forward and backward selection methods were used. In each forward step, the independent variable with the smallest probability of F not in the regression equation and ≤0.05 was included. In each backward step, the independent variable with a probability of F ≥0.10 was removed. The analysis ended if no variables met the criteria for inclusion or exclusion. A *post hoc* statistical power was conducted using G*Power software (ver. 3.1.9.2; Heinrich-Heine-Universität Düsseldorf) and MATLAB (MathWorks Inc., Natick, MA, USA). The significance level was set at *p* = 0.05 and we estimated effect sizes using partial eta squared (η_p_^2^), eta squared (η^2^), r, and Cohen’s d. Statistical analyses were performed using SPSS ver. 24 (IBM-SPSS Inc., Chicago, IL, USA).

In our study, we initially conducted a sample size calculation using G*Power 3.1.9.2. We assumed a multiple regression model with 22 predictors and aimed to estimate the partial regression coefficients. With an effect size of *f* ^2^ = 0.18, corresponding to an adjusted coefficient of determination *R* ^2^ ≈ 0.15, we performed our tests at a 5% significance level and aimed for 80% power. This led us to a required sample size of 140 participants in total.

## 3 Results

The common and differing results of the parameters related to gait for patients with LHD and RHD are compiled in the Supplementary Material Data Sheet, which also includes a summary due to the extensive amount of results for ease of reference.

The *post hoc* power analysis demonstrates that despite our sample size being less than the initially estimated 140 participants, the actual power achieved with our sample of 32 patients with right-sided brain lesions, 38 patients with left-sided brain lesions, and 20 healthy controls was 0.99. This high power indicates that our study was adequately powered to detect significant effects, despite the smaller sample size.

### 3.1 Subject characteristics

We did not find significant differences in gender, age, height, weight, diagnosis, and time since stroke among the groups. The speech (*p* < 0.001, *r* = 3.15) and finger function (*p* = 0.030, *r* = 0.56) item scores in patients with LHD were lower than those with RHD. No significant difference in the other items of SIAS was also found between patients with RHD and LHD.

### 3.2 Differences in gait parameters

Tables 2–4 present the representative gait, kinetic and kinematic, and PCA-related parameters, respectively, of patients with RHD, LHD, and also healthy controls. We presented the results of the statistical power in Supplementary Tables S6–S9 as Supplementary Material.

**TABLE 2:** Mean and standard deviation of spatiotemporal and WBAM_R_ data in patients following stroke and healthy controls.

	Right-sided brain lesion		Left-sided brain lesion		Healthy controls		Two-way ANOVA					
							Right-sided brain lesion vs. controls			Left-sided brain lesion vs. controls		
							*p*-value			*p*-value		
	Paretic side	Nonparetic side	Paretic side	Nonparetic side	Right	Left	Subjects	Laterality	Interaction	Subjects	Laterality	Interaction
Gait speed (m/s)	0.52 ± 0.26		0.46 ± 0.27		0.48 ± 0.18							
Gait cycle time (s)	1.52 ± 0.47		1.67 ± 0.62		1.82 ± 0.52							
Stride length (m)	0.70 ± 0.27^a^		0.65 ± 0.27^a^		0.88 ± 0.17^a^							
Step width (m)	0.16 ± 0.05		0.16 ± 0.04		0.14 ± 0.03							
WBAM_R_	**0.13 ± 0.10**		**0.16 ± 0.13** ^b^		**0.08 ± 0.04** ^b^							
Stance time (s)	0.96 ± 0.43^c^ ^,^ ^d^	1.17 ± 0.51^c^	1.14 ± 0.61^c^	1.35 ± 0.68^c^	1.25 ± 0.45	1.23 ± 0.43^d^	0.186	*<*0.000	*<*0.000	0.998	*<*0.000	*<*0.000
Swing time (s)	0.55 ± 0.10	0.37 ± 0.09	0.54 ± 0.10	0.38 ± 0.14	0.58 ± 0.10	0.59 ± 0.10	*<*0.000	*<*0.000	*<*0.000	*<*0.000	0.002	0.001
Step length (m)	0.36 ± 0.12^c^ ^,^ ^d^	0.32 ± 0.15^c^ ^,^ ^e^	0.33 ± 0.13^c^ ^,^ ^f^	0.31 ± 0.15^b^ ^,^ ^c^	0.43 ± 0.09^e^ ^,^ ^f^	0.43 ± 0.08^b^ ^,^ ^d^	0.008	0.030	0.146	0.001	0.500	0.140

^a^ Significantly different between patients and healthy controls at *p* < 0.05.

^b^ Significantly different between the left sides in patients with left-sided brain lesion and healthy controls at *p* < 0.05.

^c^ Significantly different between the paretic and nonparetic sides in patients at *p* < 0.05.

^d^ Significantly different between the left sides in patients with right-sided brain lesion and healthy controls at *p* < 0.05.

^e^ Significantly different between the right sides in patients with right-sided brain lesion and healthy controls at *p* < 0.05.

^f^ Significantly different between the right sides in patients with left-sided brain lesion and healthy controls at *p* < 0.05.

**TABLE 3:** Mean and standard deviation of kinematic and kinetic data in patients following stroke and healthy controls.

	Right-sided brain lesion		Left-sided brain lesion		Healthy controls		Two-way ANOVA					
							Right-sided brain lesion vs. controls			Left-sided brain lesion vs. controls		
							*p*-value			*p*-value		
	Left side	Right side	Right side	Left side	Right side	Left side	Subjects	Laterality	Interaction	Subjects	Laterality	Interaction
	Paretic side	Nonparetic side	Paretic side	Nonparetic side								
Peak hip extension moment in early stance (Nm/kg)	0.42 ± 0.30^a^	0.61 ± 0.34^a^ ^,^ ^b^	0.34 ± 0.26^a^ ^,^ ^c^	0.46 ± 0.21^a^ ^,^ ^c^ ^,^ ^d^	0.29 ± 0.10^b^	0.31 ± 0.15^d^	0.004	0.010	0.002	0.066	0.004	0.031
Peak hip flexion moment in the stance phase (Nm/kg)	0.57 ± 0.33	0.62 ± 0.28	0.64 ± 0.32^a^	0.54 ± 0.26^a^	0.68 ± 0.28	0.61 ± 0.17	0.498	0.140	0.835	0.412	0.038	0.687
First peak knee extension moment in the stance phase (Nm/kg)	0.16 ± 0.14^a^ ^,^ ^e^	0.33 ± 0.20^a^ ^,^ ^b^	0.31 ± 0.21^e^	0.34 ± 0.26^d^	0.22 ± 0.17^b^	0.18 ± 0.15^d^	0.199	0.003	0.051	0.019	0.887	0.240
Peak knee flexion moment in the stance phase (Nm/kg)	0.37 ± 0.32^a^ ^,^ ^e^ ^,^ ^f^	−0.04 ± 0.17^a^ ^,^ ^b^	0.12 ± 0.27^a^ ^,^ ^e^	−0.01 ± 0.20^a^ ^,^ ^d^	0.15 ± 0.13^b^	0.18 ± 0.11^d^ ^,^ ^f^	0.968	<0.000	<0.000	0.011	0.226	0.047
Second peak knee extension moment in the stance phase (Nm/kg)	0.19 ± 0.16^a^ ^,^ ^e^	0.44 ± 0.25^a^ ^,^ ^b^	0.34 ± 0.23^a^ ^,^ ^e^	0.47 ± 0.30^a^ ^,^ ^d^	0.32 ± 0.14^b^	0.25 ± 0.08^d^	0.383	<0.000	0.013	0.025	0.479	0.004
Peak ankle dorsiflexion moment in early stance (Nm/kg)	0.03 ± 0.07^a^ ^,^ ^f^	0.09 ± 0.09^a^	0.03 ± 0.06^a^ ^,^ ^c^ ^,^ ^g^	0.07 ± 0.05^a^ ^,^ ^c^	0.07 ± 0.05^g^	0.07 ± 0.06^f^	0.626	0.016	0.012	0.093	0.175	0.221
Peak ankle plantarflexion moment in the stance phase (Nm/kg)	0.72 ± 0.27^a^ ^,^ ^f^	0.95 ± 0.20^a^	0.68 ± 0.30^a^ ^,^ ^g^	0.89 ± 0.24^a^	0.96 ± 0.17^g^	1.00 ± 0.16^f^	0.008	0.001	<0.000	0.002	<0.000	0.002
Peak hip extension in stance (°)	−6.1 ± 7.9^a^ ^,^ ^f^	−12.8 ± 17.1^a^ ^,^ ^b^	−8.9 ± 8.9^a^ ^,^ ^g^	−12.7 ± 14.2^a^ ^,^ ^d^	1.6 ± 6.5^b^ ^,^ ^g^	1.7 ± 6.5^f^ ^,^ ^d^	<0.000	0.035	0.043	<0.000	0.290	0.255
Peak hip flexion in early stance (°)	26.1 ± 7.1	23.5 ± 15.5	28.9 ± 8.0	27.7 ± 14.8	27.6 ± 4.3	27.4 ± 4.3	0.233	0.456	0.418	0.709	0.705	0.753
Peak knee flexion in early stance (°)	11.1 ± 9.9^a^ ^,^ ^e^	19.2 ± 5.8^a^ ^,^ ^b^ ^,^ ^e^	17.7 ± 9.3^a^ ^,^ ^g^	20.7 ± 8.5^a^ ^,^ ^d^	12.9 ± 5.7^b^ ^,^ ^g^	12.1 ± 5.0^d^	0.068	0.004	0.017	<0.000	0.362	0.109
Peak knee extension in stance (°)	−0.9 ± 9.0^a^ ^,^ ^e^	−8.1 ± 5.3^a^ ^,^ ^b^ ^,^ ^e^	−7.1 ± 8.2^a^	−9.5 ± 7.5^a^ ^,^ ^d^	−4.2 ± 5.3^b^	−2.2 ± 5.3^d^	0.346	0.001	0.050	0.003	0.877	0.051
Peak knee flexion in late stance (°)	22.5 ± 12.7^a^ ^,^ ^f^	44.4 ± 4.9^a^ ^,^ ^b^	27.7 ± 11.1^a^ ^,^ ^g^	43.7 ± 6.7^a^ ^,^ ^d^	35.3 ± 5.1^b^ ^,^ ^g^	34.9 ± 6.8^f^ ^,^ ^d^	0.345	<0.000	<0.000	0.718	<0.000	<0.000
Ankle plantarflexion in early stance (°)	5.4 ± 8.7^a^	−1.4 ± 4.6^a^ ^,^ ^b^	3.3 ± 5.3^a^	0.4 ± 6.3^a^	3.4 ± 3.7^b^	2.9 ± 3.1	0.342	0.010	0.003	0.189	0.096	0.254
Ankle dorsiflexion in stance (°)	11.0 ± 9.6^a^ ^,^ ^e^ ^,^ ^f^	21.2 ± 3.4^a^ ^,^ ^b^	15.1 ± 6.5^a^ ^,^ ^f^ ^,^ ^g^	20.0 ± 5.7^a^	18.9 ± 3.8^b^ ^,^ ^g^	19.2 ± 2.8^f^	0.019	<0.000	<0.000	0.168	0.012	0.024

^a^ Significantly different between the paretic and nonparetic sides in patients at *p* < 0.05.

^b^ Significantly different between the right sides in patients with right-sided brain lesion and healthy controls at *p* < 0.05.

^c^ Significantly different between the nonparetic sides in patients with left-sided and right-sided brain lesion.

^d^ Significantly different between the left sides in patients with left-sided brain lesion and healthy controls at *p* < 0.05.

^e^ Significantly different between the paretic sides in patients with left-sided and right-sided brain lesion.

^f^ Significantly different between the left sides in patients with right-sided brain lesion and healthy controls at *p* < 0.05.

^g^ Significantly different between the right sides in patients with left-sided brain lesion and healthy controls at *p* < 0.05.

**TABLE 4:** Mean and standard deviation results for the PCA-related data.

	Right-sided brain lesion		Left-sided brain lesion		Healthy controls		Two-way ANOVA					
							Right-sided brain lesion vs. controls			Left-sided brain lesion vs. controls		
							*p*-Value			*p*-Value		
	Left side	Right side	Right side	Left side	Right side	Left side	Subjects	Laterality	Interaction	Subjects	Laterality	Interaction
	Paretic side	Nonparetic side	Paretic side	Nonparetic side								
Timing of peak PC1 (% stance phase)	67.22 ± 16.45^a^ ^,^ ^b^	75.28 ± 14.67^a^	68.74 ± 15.92^c^	54.40 ± 34.15^d^	79.15 ± 6.75^c^	81.35 ± 6.86^b^ ^,^ ^d^	0.170	0.227	0.037	*<* 0.000	0.163	0.059
Variance explained by PC1 (%)	0.78 ± 0.12	0.77 ± 0.12	0.81 ± 0.10^a^	0.74 ± 0.13^a^	0.79 ± 0.10	0.78 ± 0.08	0.631	0.766	0.663	0.550	0.063	0.086
Variance explained by PC2 (%)	0.20 ± 0.10	0.18 ± 0.10	0.17 ± 0.10	0.22 ± 0.11	0.20 ± 0.09	0.19 ± 0.08	0.879	0.712	0.616	0.950	0.224	0.192
Variance explained by PC1 + PC2 (%)	0.98 ± 0.03^a^	0.95 ± 0.04^a^ ^,^ ^e^	0.98 ± 0.02^a^	0.95 ± 0.04^a^ ^,^ ^d^	0.98 ± 0.02^e^	0.98 ± 0.02^d^	0.027	0.035	0.005	0.046	0.006	0.055
Loadings of ankle joint moment in PC1	0.66 ± 0.15	0.62 ± 0.09	0.66 ± 0.11^a^ ^,^ ^c^	0.61 ± 0.16^a^	0.60 ± 0.07^c^	0.63 ± 0.07	0.125	0.163	0.763	0.376	0.421	0.037
Loadings of knee joint moment in PC1	−0.23 ± 0.51^a^ ^,^ ^b^ ^,^ ^f^	0.37 ± 0.45^a^	0.27 ± 0.49^f^	0.38 ± 0.45	0.28 ± 0.53	0.23 ± 0.51^b^	0.122	*<* 0.000	0.001	0.541	0.720	0.307
Loadings of hip joint moment in PC1	−0.22 ± 0.44^a^ ^,^ ^b^	−0.39 ± 0.34^a^	−0.38 ± 0.32^c^	−0.32 ± 0.41^d^	−0.52 ± 0.15^c^	−0.54 ± 0.10^b^ ^,^ ^d^	0.005	0.127	0.058	0.012	0.638	0.379
Loadings of ankle joint moment in PC2	0.15 ± 0.38^a^ ^,^ ^b^ ^,^ ^f^	−0.25 ± 0.42^a^	−0.16 ± 0.41^f^	−0.12 ± 0.47	−0.19 ± 0.41	−0.19 ± 0.41^b^	0.137	0.005	0.005	0.616	0.779	0.765
Loadings of knee joint moment in PC2	0.57 ± 0.28^b^	0.50 ± 0.31^e^	0.60 ± 0.23^a^	0.47 ± 0.30^a^	0.68 ± 0.24^c^ ^,^ ^g^	0.73 ± 0.20^b^ ^,^ ^g^	0.005	0.253	0.860	0.004	0.253	0.017
Loadings of hip joint moment in PC2	−0.38 ± 0.56^a^ ^,^ ^b^	0.11 ± 0.65^a^	−0.10 ± 0.63	0.04 ± 0.69	0.10 ± 0.54	0.05 ± 0.49^b^	0.107	0.010	0.038	0.419	0.677	0.357

^a^ Significantly different between the paretic and nonparetic sides in patients at *p* < 0.05.

^b^ Significantly different between the left sides in patients with right-sided brain lesion and healthy controls at *p* < 0.05.

^c^ Significantly different between the right sides in patients with left-sided brain lesion and healthy controls at *p* < 0.05.

^d^ Significantly different between the left sides in patients with left-sided brain lesion and healthy controls at *p* < 0.05.

^e^ Significantly different between the right sides in patients with right-sided brain lesion and healthy controls at *p* < 0.05.

^f^ Significantly different between the paretic sides in patients with left-sided and right-sided brain lesion.

^g^ Significantly different between the right and left sides in healthy controls at *p* < 0.05.

#### 3.2.1 Gait speed, step length, gait cycle time, step width, and WBAM

No significant differences were observed in gait cycle time, gait speed, location of lesion, and step width among the three groups. However, significant differences were found in stride length (F_(2,87)_ = 5.611, *p* = 0.005, η^2^ = 0.11) and WBAM_R_ (F_(2,87)_ = 3.288, *p* = 0.042, η^2^ = 0.07). The stride lengths of patients with RHD (*p* = 0.004, *d* = 0.95) and LHD (*p* = 0.034, *d* = 0.77) were shorter than those of healthy controls. There was no significant difference in WBAM_R_ between patients with RHD and LHD. However, the WBAM_R_ in patients with LHD was larger than that of healthy controls (*p* = 0.032, *d* = 0.68) (Table 2).

#### 3.2.2 Spatiotemporal parameters in patients with RHD

Significant main effects of group (F_(1,50)_ = 7.654, *p* = 0.008, η_p_^2^ = 0.13) and side (F_(1,50)_ = 4.981, *p* = 0.030, η_p_^2^ = 0.09) on step length were found. Patients with RHD had shorter step lengths than controls, and the step length on the left side was longer than on the right side (Table 2).

Significant main effects of group (F_(1,50)_ = 33.112, *p* < 0.001, η_p_^2^ = 0.40), side (F_(1,50)_ = 29.223, *p* < 0.001, η_p_^2^ = 0.37), and interactions between group and side (F_(1,50)_ = 28.013, *p* < 0.001, η_p_^2^ = 0.36) were also found on swing time. The swing time on the paretic (left) side was longer than on the nonparetic (right) side in patients with RHD (*p* < 0.001, *d* = 1.94). The swing time on the nonparetic (right) side in patients with RHD was shorter than that on the right side in healthy controls (*p* < 0.001, *d* = 2.33).

In addition, there were significant main effects of side (F_(1,50)_ = 33.626, *p* < 0.001, η_p_^2^ = 0.40) and interaction between group and side (F_(1,50)_ = 25.687, *p* < 0.001, η_p_^2^ = 0.34) on stance time. The *post hoc* test showed that the stance time was shorter on the paretic (left) side than on the nonparetic (right) side in patients with RHD (*p* < 0.001, *d* = 0.44). The stance time was shorter on the paretic (left) side in patients with RHD than on the left side in healthy controls (*p* = 0.031, *d* = 0.63).

#### 3.2.3 Spatiotemporal parameters in patients with LHD

Significant main effects of group (F_(1,50)_ = 11.766, *p* = 0.001, η_p_^2^ = 0.17) on step length, with patients with LHD having shorter step lengths than the controls, were found (Table 2).

There were also significant main effects of group (F_(1,50)_ *=* 32.147, *p <* 0.001, η_p_^2^ = 0.37)*,* side (F_(1,50)_ = 11.030, *p* = 0.002, η_p_^2^ = 0.17), and interactions between group and side (F_(1,50)_ = 12.974, *p* = 0.001, η_p_^2^ = 0.19) on swing time. The swing time on the paretic (right) side was longer than that on the nonparetic (left) side in patients with LHD (*p* < 0.001, *d* = 1.29). The swing time on the nonparetic (left) side was shorter than that on the left side in healthy controls (*p* < 0.001, *d* = 1.60).

Furthermore, there were significant main effects of side (F_(1,50)_ *=* 15.989*, p <* 0.001*,* η_p_^2^ *=* 0.22) and interaction between group and side (*F*_(1,50)_ = 20.884, *p* < 0.001, η_p_^2^ = 0.27) on stance time. The *post hoc* test found that the stance time was shorter on the paretic (right) side than on the nonparetic (left) side in patients with LHD (*p* < 0.001, *d* = 0.32).

#### 3.2.4 Kinetic and kinematic parameters of patients with RHD

Significant main effects of side and interaction between group and side were observed on all kinetic parameters, except the peak hip flexion moment in the stance phase, as presented in Table 3. Moreover, the results showed significant main effects of group on the peak hip extension moment in early stance (F_(1,50)_ = 9.307, *p* = 0.004, η_p_^2^ = 0.16) and peak ankle plantarflexion moment in the stance phase (F_(1,50)_ = 7.599, *p* = 0.008, η_p_^2^ = 0.13). In patients with RHD, both the first (*p* < 0.001, d = 0.81) and second peak knee extension moments (*p <* 0.001, *d =* 0.76) on the paretic side during the stance phase were smaller than those in patients with LHD. The peak knee flexion moment during the stance phase on the paretic side was larger in patients with RHD than in those with LHD (*p <* 0.001, *d =* 0.86). Furthermore, the peak hip extension moment (*p =* 0.033, d = 0.52) and ankle dorsiflexion (*p =* 0.048, *d =* 0.48) in early stance on the nonparetic side were larger in patients with RHD than in those with LHD (Table 3).

The results of the *post hoc* test for kinetic parameters were as follows. The peak hip extension moment and first and second peak knee extension moments in stance on the nonparetic (right) side in patients with RHD were larger than those on the right side in healthy controls (*p* < 0.001, *d* = 1.13; *p* = 0.041, *d* = 0.60; *p* = 0.049, *d* = 0.58) and those on the paretic (left) side in patients with RHD (*p* < 0.001, *d* = 0.53; *p* < 0.001, *d* = 0.98; *p* < 0.001, *d* = 1.23). Although the peak knee flexion moment in the stance phase on the nonparetic (right) side was smaller than that on the paretic (left) side (*p* < 0.001, *d* = 1.59) and that on the right side in healthy controls (*p* < 0.001, *d* = 1.24), the peak knee flexion moment in the stance phase on the paretic (left) side in patients was larger than that on the left side in healthy controls (*p* = 0.015, *d* = 0.72). The peak ankle dorsiflexion moment in early stance and ankle plantarflexion moment in the stance phase on the paretic (left) side were smaller than those on the nonparetic (right) side in patients (*p* < 0.001, *d* = 0.76 and *p* < 0.001, *d* = 0.98) and those on the left side in healthy controls (*p* = 0.043, *d* = 0.59 and *p* < 0.001, *d* = 1.22).

There were significant main effects of group, side, and interaction between group, and side on kinematics such as the peak hip extension in stance, peak knee flexion in early stance, and peak ankle dorsiflexion in stance, as presented in Table 3. In addition, there were significant main effects of side and interaction between group and side on the peak knee extension in stance, peak knee flexion in late stance, and peak ankle plantarflexion in early stance. The peak knee flexion (*p =* 0.005, *d =* 0.69) and peak ankle dorsiflexion (*p =* 0.037, *d =* 0.51) in early stance on the paretic side in patients with RHD were smaller than those with LHD. On the other hand, the peak knee extension in stance on the paretic side with RHD was larger than those with LHD (*p =* 0.004, *d =* 0.72).

The results of the *post hoc* test for kinematic parameters were as follows. The peak hip extension in the stance phase, peak knee extension in the stance phase, and peak ankle plantarflexion in early stance on the nonparetic (right) side were lower than those on the paretic (left) side (*p* = 0.001, *d* = 0.59; *p* < 0.001, *d* = 0.97; *p* < 0.001, *d* = 0.98) and those on the right side (*p* = 0.001, *d* = 1.06; *p* = 0.013, *d* = 0.74; *p* < 0.001, *d* = 1.12) in healthy controls. The peak knee flexion in early and late stance and peak ankle dorsiflexion in the stance phase on the nonparetic side (right) were higher than those on the paretic (left) side in patients (*p* < 0.001, *d* = 1.01; *p* < 0.001, *d* = 2.27; *p* < 0.001, *d* = 1.41) and those on the right side in healthy controls (*p* < 0.001, *d* = 1.09; *p* < 0.001, *d* = 1.82; *p* = 0.030, *d* = 0.64). The peak hip extension in stance, peak knee flexion in late stance, and peak ankle dorsiflexion in stance on the paretic (left) side were also smaller than those on the left side in healthy controls (*p* = 0.001, *d* = 1.02; *p* < 0.001, *d* = 1.14; *p* = 0.001, *d* = 1.05).

#### 3.2.5 Kinetic and kinematic parameters of patients with LHD

A significant main effect of side on kinetics such as the peak hip flexion moment in the stance phase was observed, which was larger on the right side than on the left side. Significant main effects of group on the first peak knee extension moment in the stance phase and peak ankle dorsiflexion moment in early stance were also observed, which were larger in patients than in healthy controls. There were significant main effects of group and interaction between group and side on the peak knee flexion in the stance phase and second peak knee extension moment in the stance phase. Moreover, significant main effects of group, side, and interaction between group, and side on the peak ankle plantarflexion moment in the stance phase were found (Table 3).

The results of the *post hoc* test for kinetic parameters were as follows. The peak hip extension moment and second peak knee extension moment in stance on the nonparetic (left) side in patients with RHD were larger than those on the left side in healthy controls (*p* = 0.006, *d* = 0.79 and *p* = 0.003, *d* = 0.48) and those on the paretic (right) side in patients (*p* < 0.001, *d* = 0.53 and *p* = 0.002, *d* = 0.90). The peak knee flexion moment in the stance phase on the nonparetic (left) side in patients with RHD was smaller than that on the left side in healthy controls (*p* < 0.001, *d* = 1.16) and that on the paretic (right) side in patients (*p* = 0.007, *d* = 0.52). The peak ankle plantarflexion moment in the stance phase on the paretic (right) side was smaller than that on the nonparetic (left) side in patients (*p* < 0.001, *d* = 0.78) and that on the left side in healthy controls (*p* < 0.001, *d* = 1.08).

Significant main effects of side and interaction between group and side were observed on kinematics such as the peak knee flexion in the late stance phase and peak ankle dorsiflexion in the stance phase, as presented in Table 3. In addition, there were significant main effects of group and interaction between group and side on the peak knee extension in stance. Significant main effects of side on the peak ankle plantarflexion in early stance were found, which was larger on the right side than on the left side. Significant main effects of group on the peak hip extension and peak knee flexion in early stance were also observed, which were smaller, and larger, respectively, in patients than in healthy controls.

The results of the *post hoc* test for kinematic parameters were as follows. The peak knee extension in the stance phase on the nonparetic (left) side in patients was smaller than that on the left side in healthy controls (*p* < 0.001, *d* = 1.07). The peak knee flexion in the late stance phase on the paretic (right) side in patients was smaller than those on the nonparetic (left) side (*p* < 0.001, *d* = 1.74) and on the right side in healthy controls (*p* = 0.005, *d* = 0.80). The peak knee flexion in late stance on the nonparetic side (right) was higher than that on the right side in healthy controls (*p* < 0.001, *d* = 1.31).

#### 3.2.6 PCA-related parameters of patients with RHD

There were significant main effects of group on the peak timing of the first PC (F_(1,50)_ = 10.234, *p* = 0.002, η_p_^2^ = 0.17) and the percent variance of the first two PCs (F_(1,50)_ = 5.183, *p* = 0.027, η_p_^2^ = 0.09) as well as of interaction between group and side on the peak timing of the first PC (F_(1,50)_ = 4.581, *p* = 0.037, η_p_^2^ = 0.08) and the percent variance of the first two PCs (F_(1,50)_ = 8.837, *p* = 0.005, η_p_^2^ = 0.15). A significant main effect of side was observed on the percent variance of the first two PCs (F_(1,50)_ = 4.678, *p* = 0.035, η_p_^2^ = 0.09). The *post hoc* test indicated earlier peak timing of the first PC on the paretic (left) side than on the nonparetic (right) side (*p* = 0.009, *d* = 0.52) and the left side in healthy controls (*p* = 0.001, *d* = 1.04). The percent variance of the first two PCs on the nonparetic (right) side was lower than those on the paretic (left) side (*p* < 0.001, *d* = 0.85) and on the right side in healthy controls (*p* = 0.002, *d* = 0.94) (Table 4).

There were significant main effects of side on the loading of knee joint moment for the first PC (F_(1,50)_ = 17.572, *p* < 0.001, η_p_^2^ = 0.26), of group on the loading of hip joint moment for the first PC (F_(1,50)_ = 8.636, *p* = 0.005, η_p_^2^ = 0.15), and of interaction between group and side on the loading of knee joint moment for the first PC (F_(1,50)_ = 12.488, *p* = 0.001, η_p_^2^ = 0.20). The *post hoc* test showed that the loading of hip joint moment for the first PC was a negative value and larger in patients than in healthy controls. On the paretic (left) side, the loading of knee joint moment for the first PC was a negative value and lower than those on the nonparetic (right) side (*p* < 0.001, *d* = 1.26) and on the left side in healthy controls (*p* = 0.003, *d* = 0.90). The loading of knee joint moment for the first PC on the paretic side in patients with RHD was smaller than those with LHD (*p* < 0.001, *d* = 1.01).

#### 3.2.7 PCA-related parameters of patients with LHD

There were significant main effects of group on the peak timing of the first PC (F_(1,50)_ = 18.800, *p* < 0.001, η_p_^2^ = 0.25) and the percent variance of the first two PCs (F_(1,50)_ = 4.169, *p* = 0.046, η_p_^2^ = 0.07) as well as of side on the percent variance of the first two PCs (F_(1,50)_ = 8.097, *p* = 0.006, η_p_^2^ = 0.13). There seemed to be an interaction between group and side in the percent variance of the first two PCs (F_(1,50)_ = 3.828, *p* = 0.055, η_p_^2^ = 0.06). The *post hoc* test indicated earlier peak timing of the first PC on the paretic (right) and nonparetic (left) sides in patients than on the right (*p* = 0.007, *d* = 0.77) and left (*p* = 0.008, *d* = 0.76) sides in healthy controls. The percent variance of the first two PCs on the nonparetic (left) side was lower than those on the paretic (right) side (*p* < 0.001, *d* = 0.78) and the left side of healthy controls (*p* = 0.023, *d* = 0.64) (Table 4).

There were significant main effects of the group on the loading of hip joint moment for the first PC (F_(1,50)_ = 6.723, *p* = 0.012, η_p_^2^ = 0.11) as well as of interaction between group and side on the loading of ankle joint moment for the first PC (F_(1,50)_ = 4.571, *p* = 0.037, η_p_^2^ = 0.08). The *post hoc* test indicated that the loading of hip joint moment for the first PC was a negative value and larger in patients than in healthy controls. The loading of ankle joint moment for the first PC was higher on the paretic (right) side than on the nonparetic (left) side (*p* = 0.015, *d* = 0.42) and right side in healthy controls (*p* = 0.016, *d* = 0.69).

### 3.3 Influence of kinetic and PCA-Related parameters on walking speed

The results obtained from the multiple regression analysis of gait speed for patients with RHD and LHD lesions and healthy controls are presented in Table 5. In patients with LHD and healthy controls, the peak ankle dorsiflexion moment in early stance on the left side (nonparetic side) and timing of peak PC1 on the right side (paretic side) were found to be significant gait speed predictors. Furthermore, only in patients with LHD, the peak ankle plantarflexion moment in the stance phase on the right side (paretic side) found to be a significant gait speed predictor. In healthy controls, the peak hip flexion moment in the stance phase on the left side was found to contribute to gait speed. In patients with RHD, the peak ankle dorsiflexion moment in early stance and ankle plantarflexion moment in the stance phase on the right side (nonparetic side), as well as ankle plantarflexion moment in the stance phase on the left side (paretic side), were identified as contributors to gait speed. The statistical power in the multiple regression analysis across all groups was 0.99, indicating that the findings are likely reflecting actual effects rather than being products of chance (Table 5).

**TABLE 5:** Multiple regression analysis with gait speed in patients with hemiparesis and healthy controls.

Variable	Partial regression coefficient	Standardized partial regression coefficient	Variance, *R* ^2^	*p*-value	VIF
Healthy controls					
Peak ankle dorsiflexion moment in early stance on the left side	−1.640	−0.528	0.565	0.001	1.391
Timing of peak PC1 on the right side	0.011	0.402	0.735	0.002	1.051
Peak hip flexion moment in the stance phase on the right side	−0.091	−0.304	0.798	0.023	1.380
Patients with right-sided brain lesion					
Peak ankle plantarflexion moment in the stance phase on the paretic side	0.414	0.428	0.559	0.001	1.596
Peak ankle plantarflexion moment in the stance phase on the nonparetic side	0.495	0.380	0.662	0.002	1.475
Peak ankle dorsiflexion moment in early stance on the nonparetic side	−0.904	−0.310	0.741	0.004	1.165
Patients with left-sided brain lesion					
Peak ankle plantarflexion moment in the stance phase on the paretic side	0.506	0.548	0.517	0.000	1.147
Peak ankle dorsiflexion moment in early stance on the nonparetic side	−2.040	10.423	0.682	0.000	1.101
Timing of peak PC1 on the paretic side	0.004	0.233	0.728	0.012	1.054

^a^ PC, principal component.

## 4 Discussion

The factors that contributed to gait speed varied among patients with RHD, patients with LHD, and controls. Consistent with hypothesis, in patients with RHD, larger ankle plantarflexion moments on both the paretic (left) and nonparetic (right) sides as well as ankle dorsiflexion moment on the nonparetic (right) side were contributing factors. However, contrary to hypothesis, WBAM in RHD did not differ from that in patient with LHD and healthy controls. The results may reflect the cautious gait in patients with RHD, who have instability while standing. In patients with LHD, larger ankle plantarflexion moment and delayed peak timing of the first PC on the paretic (right) side as well as ankle dorsiflexion moment on the nonparetic (left) side were contributing factors. In controls, larger ankle dorsiflexion moment as well as larger hip flexion moment on the left side and delayed peak timing of the first PC on the right side were contributing factors. Contrary to hypothesis, our results indicated that patients with LHD controlled walking speed by the timing of kinetic coordination on the paretic (right) side, similar to control groups. No study has investigated the factors contributing to gait speed in patients with stroke by simultaneously including kinetic parameters of both the paretic and nonparetic sides. To the best of our knowledge, this is the first study that included bilateral kinetic factors in a multiple regression analysis and demonstrated that the kinetic factors contributing to gait speed differ between patients with LHD and RHD.

Our findings suggested that, much like the control groups, patients with LHD managed their walking speed by coordinating the timing of kinetic movements on their paretic (right) side. Previous research has shown that left hemisphere dominance for skilled movement is attributed to anatomical and functional hemispheric asymmetries of the primary motor cortex, descending pathways, and somatosensory association and premotor cortices (Serrien et al., 2006). Indeed, patients with a left hemisphere lesion performing an upper-limb task demonstrated a deficit in intersegmental coordination (Schaefer et al., 2012). Additionally, our study observed a more pronounced impairment of motor function and language abilities in the paretic (right) hand of patients with left hemisphere damage (LHD) compared to those with right hemisphere damage (RHD). This observation may be attributed to the lateralization of brain function, where the left hemisphere, typically dominant in right-handed individuals, is primarily responsible for tasks involving language and fine motor skills. These findings suggest that the significant relationship between gait speed and the timing of kinetic coordination on the right side in controls and patients with LHD may be due to left hemisphere dominance. Furthermore, previous studies have demonstrated that the processing of sensory-motor data is carried out by a more extensive and densely connected network in the dominant left hemisphere (Guye et al., 2003). Therefore, damage to one network component is more easily compensated for by other network components, indicating that patients with LHD could control gait speed by timing kinetic coordination despite a left hemisphere lesion.

In healthy controls, gait speed was associated with the timing of peak PC1 on the right side and hip flexion moment and ankle dorsiflexion moment on the left side. Healthy controls with delayed timing of peak PC1 on the right side had faster gait speed, consistent with a previous study (Sekiguchi et al., 2019). Furthermore, hip flexion moment was involved in gait speed, consistent with another previous study (Fukuchi et al., 2019). Hip flexion moment may also be involved in the propulsion of the lower limb during the swing phase. The involvement of the ankle dorsiflexion moment on the left side may be due to the influence of the heel rocker function during gait. The period when the peak ankle dorsiflexion moment on the left side was observed is the early stance phase on the left side and the late stance phase on the right side, respectively. During this period, as walking speed increases due to increased propulsion of the lower limb on the right side, the left lower limb may be possibly stabilized by an appropriate ankle dorsiflexion moment on the left side to break the ankle plantarflexion movement. Patients with lesion in the right hemisphere had decreased ability to shift body weight as well as poorer body sway and stance control, indicating that the right hemisphere may be associated with stability (Fernandes et al., 2018; Coelho et al., 2019). These facts support the idea that as walking speed increases, the ankle dorsiflexion moment is controlled by the right hemisphere to stabilize the left lower limb, whereas the left hemisphere is involved in skilled movements such as intersegmental coordination.

In patients with LHD, gait speed was associated with left ankle dorsiflexion moment and right PC1 timing. In these patients, the factors involved in gait speed were similar to those in healthy controls, except for the plantarflexion moment of the paretic (right) ankle. Contrary to patients with RHD, the right hemisphere involved in stability was not damaged and was able to maintain balance control in the paretic lower limb, such as increased knee flexion and decreased hip extension angle in early stance and increased knee extension moment which is support moment in patients with LHD. This may be a factor in performing similar kinetic control as healthy individuals with similar gait speed. Consistent with the previous study, the timing of peak PC1 was earlier on the paretic side (Sekiguchi et al., 2022). Like in other studies on muscle synergy, the timing of the impaired motor module involved in paretic propulsion may be involved in gait speed (Routson et al., 2013). The hip flexion moment was greater in the paretic (right) than in the nonparetic (left) side in patients with LHD, similar to healthy subjects. However, the plantarflexion moment was reduced on the paretic (right) side. Thus, it is possible that the impaired plantarflexion moment on the paretic side had a greater effect on gait speed, similar to the result of a previous study (Olney et al., 1994).

The absolute values of the loadings of ankle and hip joint moments for the first PC were high in both patients and healthy controls. The first motor module, which comprised of ankle, and hip joint moments, plays a role in inducing propulsion and supporting weight (Sadeghi et al., 2001). In patients with RHD, the loading of knee joint moment for the first PC on the paretic (left) side was a negative value, representing flexion moment, which is unlike the patients with LHD. A previous study showed excessive cocontraction of ankle plantar flexors and knee flexors in the stance phase during gait in patients with stroke (Fujita et al., 2018). In addition, patients with RHD had larger knee flexion moments in the stance phase on the paretic (left) side than those with LHD and in healthy controls. These facts indicate that the first motor module of kinetic variables merged with the knee flexion moment due to excessive cocontraction of knee flexor and ankle plantar flexor on the paretic (left) side in patients with RHD. As the knee flexion moment decreases the support moment, patients with RHD have controls that reduce both the quantity and quality of the support moments in the stance phase on the paretic side during gait. Kinetic control by kinetic coordination on the paretic side in patients with RHD may cause lower-limb instability on the paretic side and larger knee extension and smaller ankle dorsiflexion in the stance phase on the paretic side to increase stability. Hip extension on the nonparetic side was decreased to prevent instability in the stance phase on the paretic (left) side by taking shorter steps like caution gait (Eils et al., 2004). Additionally, the stance time on the paretic (left) side in patients with RHD was shorter than that in controls. However, this was not observed on the paretic (right) side in patients with LHD. The impulse, calculated by multiplying the stance time by the ground reaction force, influences angular momentum. The reduced stance time could explain why the WBAM_R_ in patients with RHD did not differ from that in healthy controls. This may also be indicative of a cautious gait pattern. A decrease in hip extension on the nonparetic side, a component of trail limb angle that contributes to propulsion, relatively increases the contribution of ankle plantarflexion moment on the nonparetic side to gait speed (Hsiao et al., 2015; Hsiao et al., 2016a).

Patients with RHD had different results in kinetic factors contributing to gait speed from healthy subjects and patients with LHD. In patients with RHD, the kinetic factors included dorsiflexion moment in the early stance on the nonparetic (right) side and plantarflexion moment in the late stance on the paretic (left) side, consistent with those in patients with LHD. The results of this study indicate that kinetic control by ankle plantarflexion moment on the paretic side, which induces propulsion (Hsiao et al., 2015; Hsiao et al., 2016a), and ankle dorsiflexion moment on the nonparetic side, which controls ankle plantarflexion and braking, in the late stance in patients with stroke is important for increasing gait speed. However, from a left–right perspective, the kinetic factors in patients with left-sided brain lesions differed from those in patients with right-sided brain lesions. Contrary to normal subjects and patients with left-sided lesions, in patients with right-sided brain lesions, the left lower limb is responsible for propulsion, whereas the right lower limb is responsible for braking and stability. In fact, in patients with right-sided brain lesions, the timing of peak PC1 was not related to gait speed as the right limb is responsible for stability. This suggests that after stroke onset, patients with right-sided brain lesions may alternate kinetic roles of the lower limbs in gait speed between the left and right limbs.

」This study has several limitations. First, there were differences in finger motor dysfunction and language dysfunction between the left and right lesion sides. These differences may reflect dominant and nondominant hemispheric effects due to the lesion side. Finger motor dysfunction may affect the lower-limb kinetic variables and gait speed, consistent with the result of a previous study indicating that changes in finger spasticity following botulinum toxin treatment were associated with changes in stride (Lee et al., 2023). In this study, the differences in multiple regression analysis results due to the variance between the left and right brain lesion sides may be influenced by differences caused by finger motor dysfunction rather than differences between the left and right brain lesion sides. However, because healthy controls and patients with left brain damage had similar results in multiple regression analysis, the influence of finger motor dysfunction is thought to be small. The second limitation is that this study measured barefoot walking and walked without using a cane. Therefore, it is possible that the kinetic factors of walking speed examined were different from those of daily walking. Third limitation is that we did not investigate the dominant foot. According to a previous study, about 61.6% of the general population with a broad age range is right-footed, while 8.2% is left-footed and 30.2% is mixed-footed (Tran, U. S., & Voracek, M., 2016). Since the majority of people are right-footed, it is possible that the majority of the subjects in this study were also right-footed. The fourth limitation is that due to the lack of MRI images for all cases, we were unable to perform a detailed analysis of the size and severity of the lesions in the subjects. Therefore, it is unclear whether there is difference in the size and severity of the lesions between the patients with RHD and LHD.

In conclusion, this study has provided valuable insights into the kinetic mechanisms of decreased gait speed in patient with strokes, with a specific focus on differences across brain lesion sides. For patients with right hemisphere brain damage, larger ankle plantarflexion moments on both the paretic and nonparetic sides, as well as ankle dorsiflexion moment on the nonparetic side, were significant contributors to gait speed. In contrast, for patients with left hemisphere brain damage, larger ankle plantarflexion moment and delayed peak timing of the first principal component on the paretic side, along with ankle dorsiflexion moment on the nonparetic side, were the key factors. These findings highlight the necessity of taking into account the side of the brain lesion when devising rehabilitation strategies aimed at improving gait speed in patients with stroke.

## Data Availability

The raw data supporting the conclusion of this article will be made available by the authors, with their permission, without undue reservation.
