# Supplementary material for: Differences in kinetic factors affecting gait speed between lesion sides in patients with stroke
Source: Front Bioeng Biotechnol. 2024 Mar 19;12:1240339. doi: 10.3389/fbioe.2024.1240339 (PMC10985198; doi:10.3389/fbioe.2024.1240339)
Supplement: Supplementary file 1 [file Table1.docx]

Supplemental data

**Table**. Stroke Impairment Assessment Set

|  | Right-sided brain lesion | Left-sided brain lesion |
| --- | --- | --- |
| Motor function (0/1/2/3/4/5)  U/E proximal  U/E distal *^b^*  Hip joint  Knee joint  Ankle joint  Sensory function (0/1/2/3)  U/E touch  L/E touch  U/E position  L/E position  Tone (0/1/2/3)  U/E DTRs  L/E DTRs  U/E muscle tone  L/E muscle tone  ROM (0/1/2/3)  Shoulder abduction  Ankle dorsiflexion  Pain (0/1/2/3)  Trunk (0/1/2/3)  Vertically  Abdominal MMT  Higher cortical function  Visuospatial (0/1/2/3)  Speech (0/1/2/3)*^b^*  Unaffected side function  Grip strength  Quadricep MMT | 0/14/3/7/6/2  0/9/3/6/13/1  1/5/0/6/17/3  0/0/0/13/17/2  1/5/2/7/15/2  3/5/17/7  3/7/15/7  4/5/9/14  5/5/4/18  0/6/21/5  1/9/15/7  0/6/13/2  0/6/17/9  2/8/13/9  0/15/11/6  1/7/8/16  0/2/4/26  0/1/3/28  0/0/6/26  0/1/2/29  0/0/6/26  0/0/5/27 | 2/7/15/9/3/2  5/17/1/6/6/3  0/4/4/8/19/3  0/1/5/14/15/3  5/5/5/9/10/4  3/9/17/9  0/7/18/13  6/4/9/19  2/6/4/26  2/16/14/6  1/15/15/7  0/12/13/3  0/9/21/8  1/12/13/11  4/15/13/6  1/4/13/20  0/0/4/34  0/1/7/30  0/0/4/34  0/6/14/18  0/0/11/27  0/0/5/33 |

*^a^* Values are expressed as means ± standard deviations. *^b^* Significantly different between the groups at *p* < 0.05. *^c^* The SIAS assesses neurologic impairments (upper- and lower-limb motor function, muscle tone, sensory function, range of motion, deep tendon reflexes, pain, trunk function, visuospatial function, and speech). There are 22 items, and each item is rated from 0 (severely impaired) to 3 (normal) for muscle tone, sensory function, range of motion, pain, trunk, higher cortical function, and unaffected side function or to 5 (normal) for motor function. The total score is 76. *^d^*Abbreviations: U/E, upper extremity; L/E, lower extremity; DTR, deep tendon reflex; MMT, manual muscle testing

**Table**. Placement of markers on the body

|  | Segment | Placement of markers |
| --- | --- | --- |
|  | Trunk | Spinous process of the 7th cervical vertebrae, spinous process of the 10th thoracic vertebrae, jugular notch where the clavicles meet the sternum, xiphoid process of the sternum, and the position in the middle of the right scapula |
|  | Upper arm | Both acromions and both lateral epicondyles of the elbow |
|  | Forearm | Both lateral epicondyles of the elbow and both styloid processes of the ulna and radius |
|  | Pelvis | Both anterior superior iliac spines and both posterior superior iliac spines |
|  | Thigh | Both greater trochanters and both lateral and medial epicondyles of the knee |
|  | Shank | Both lateral epicondyles of the knee and both lateral and medial malleolus |
|  | Foot | Both the first and fifth metatarsal heads, both lateral and medial malleolus, and both calcaneus |

Table 6. Statistical power in multiple regression analysis

|  | Achieved statistical power |
| --- | --- |
| Healthy controls | 0.99 |
| Patients with RHD | 0.99 |
| Patients with LHD | 0.99 |

Table 7. Statistical power in one-way ANOVA

|  | Achieved statistical power |
| --- | --- |
| Gait speed | 0.70 |
| Gait cycle time | 0.94 |
| Stride length | 0.98 |
| Step width | 0.89 |
| WBAM_R_ | 0.97 |

Table 8. Statistical power in two-way ANOVA in patients with RHD

|  | Achieved statistical power | | |
| --- | --- | --- | --- |
|  | Subjects | Laterality | Interaction |
| Stance time | 0.26 | 1.00 | 1.00 |
| Swing time | 1.00 | 1.00 | 1.00 |
| Step length | 0.77 | 0.60 | 0.31 |
| Peak hip extension moment in early stance | 0.85 | 0.75 | 0.89 |
| Peak hip flexion moment in the stance phase | 0.100 | 0.310 | 0.05 |
| First peak knee extension moment in the stance phase | 0.25 | 0.87 | 0.50 |
| Peak knee flexion moment in the stance phase | 0.05 | 1.00 | 0.99 |
| Second peak knee extension moment in the stance phase | 0.14 | 1.00 | 0.71 |
| Peak ankle dorsiflexion moment in early stance | 0.08 | 0.69 | 0.72 |
| Peak ankle plantarflexion moment in the stance phase | 0.77 | 0.93 | 1.00 |
| Peak hip extension in stance | 0.97 | 0.56 | 0.53 |
| Peak hip flexion in early stance | 0.22 | 0.11 | 0.13 |
| Peak knee flexion in early stance | 0.45 | 0.85 | 0.68 |
| Peak knee extension in stance | 0.15 | 0.94 | 0.50 |
| Peak knee flexion in late stance | 0.15 | 1.00 | 1.00 |
| Ankle plantarflexion in early stance | 0.16 | 0.75 | 0.87 |
| Ankle dorsiflexion in stance | 0.67 | 0.98 | 0.99 |
| Timing of peak PC1 (% stance phase) | 0.88 | 0.22 | 0.56 |
| Variance explained by PC1 (%) | 0.08 | 0.06 | 0.07 |
| Variance explained by PC2 (%) | 0.05 | 0.07 | 0.08 |
| Variance explained by PC1 + PC2 (%) | 0.61 | 0.56 | 0.83 |
| Loadings of ankle joint moment in PC1 | 0.33 | 0.28 | 0.06 |
| Loadings of knee joint moment in PC1 | 0.34 | 0.98 | 0.93 |
| Loadings of hip joint moment in PC1 | 0.82 | 0.33 | 0.48 |
| Loadings of ankle joint moment in PC2 | 0.32 | 0.82 | 0.82 |
| Loadings of knee joint moment in PC2 | 0.82 | 0.21 | 0.05 |
| Loadings of hip joint moment in PC2 | 0.36 | 0.75 | 0.55 |

Table 9. Statistical power in two-way ANOVA in patients with LHD

|  | Achieved statistical power | | |
| --- | --- | --- | --- |
|  | Subjects | Laterality | Interaction |
| Stance time | 0.05 | 0.98 | 0.99 |
| Swing time | 1.00 | 0.90 | 0.94 |
| Step length | 0.92 | 0.10 | 0.31 |
| Peak hip extension moment in early stance | 0.45 | 0.84 | 0.59 |
| Peak hip flexion moment in the stance phase | 0.13 | 0.55 | 0.07 |
| First peak knee extension moment in the stance phase | 0.66 | 0.05 | 0.22 |
| Peak knee flexion moment in the stance phase | 0.73 | 0.23 | 0.52 |
| Second peak knee extension moment in the stance phase | 0.62 | 0.11 | 0.84 |
| Peak ankle dorsiflexion moment in early stance | 0.39 | 0.27 | 0.23 |
| Peak ankle plantarflexion moment in the stance phase | 0.88 | 1.00 | 0.90 |
| Peak hip extension in stance | 1.00 | 0.18 | 0.20 |
| Peak hip flexion in early stance | 0.07 | 0.07 | 0.06 |
| Peak knee flexion in early stance | 0.95 | 0.15 | 0.36 |
| Peak knee extension in stance | 0.87 | 0.05 | 0.50 |
| Peak knee flexion in late stance | 0.06 | 1.00 | 1.00 |
| Ankle plantarflexion in early stance | 0.26 | 0.38 | 0.20 |
| Ankle dorsiflexion in stance | 0.28 | 0.72 | 0.62 |
| Timing of peak PC1 (% stance phase) | 0.99 | 0.28 | 0.47 |
| Variance explained by PC1 (%) | 0.09 | 0.46 | 0.40 |
| Variance explained by PC2 (%) | 0.05 | 0.21 | 0.25 |
| Variance explained by PC1 + PC2 (%) | 0.52 | 0.80 | 0.49 |
| Loadings of ankle joint moment in PC1 | 0.14 | 0.13 | 0.56 |
| Loadings of knee joint moment in PC1 | 0.09 | 0.06 | 0.17 |
| Loadings of hip joint moment in PC1 | 0.72 | 0.08 | 0.14 |
| Loadings of ankle joint moment in PC2 | 0.08 | 0.06 | 0.06 |
| Loadings of knee joint moment in PC2 | 0.83 | 0.21 | 0.67 |
| Loadings of hip joint moment in PC2 | 0.13 | 0.07 | 0.15 |
