# Supplementary material for: Differences in kinetic factors affecting gait speed between lesion sides in patients with stroke
Source: Front Bioeng Biotechnol. 2024 Mar 19;12:1240339. doi: 10.3389/fbioe.2024.1240339 (PMC10985198; doi:10.3389/fbioe.2024.1240339)
Supplement: Supplementary file 2 [file DataSheet1.docx]

**Commonalities:** Patients with RHD and LHD both showed significant group effects on the peak timing of the first Principal Component (PC) and the percent variance of the first two PCs. Additionally, both groups demonstrated larger loadings of hip joint moment for the first PC compared to healthy controls. Furthermore, both patient groups showed shorter stride lengths and step lengths compared to healthy controls.

**Characteristics of Patients with LHD:** Patients with LHD demonstrated a number of unique characteristics. The peak timing of the first PC was earlier on both the paretic and nonparetic sides compared to patients with RHD. The percent variance of the first two PCs on the nonparetic (left) side was lower in patients with LHD compared to patients with RHD. The loading of ankle joint moment for the first PC was higher on the paretic (right) side in patients with LHD compared to patients with RHD, suggesting differences in ankle joint kinetics. Additionally, patients with LHD had a larger WBAMR than healthy controls. The swing time on the paretic (right) side was longer in patients with LHD than in patients with RHD, and the stance time was shorter on the paretic (right) side in patients with LHD than in patients with RHD. In terms of kinetic parameters, a significant main effect of side on the peak hip flexion moment in the stance phase was observed, which was larger on the right side than on the left side. In terms of kinematic parameters, the peak knee extension in stance on the paretic side with RHD was larger than those with LHD.

**Characteristics of Patients with RHD:** Patients with RHD also showed distinct characteristics. They showed lower loading of knee joint moment for the first PC on the paretic (left) side compared to patients with LHD. In terms of swing time, stance time, and certain kinetic and kinematic parameters, there were distinct differences between the two patient groups. For instance, the swing time on the paretic side was longer in patients with RHD than in patients with LHD, and the stance time was shorter on the paretic side in patients with RHD than in patients with LHD. In terms of kinetic parameters, the first and second peak knee extension moments on the paretic side during the stance phase were smaller in patients with RHD than in patients with LHD. Conversely, the peak knee flexion moment during the stance phase on the paretic side was larger in patients with RHD than in patients with LHD. In terms of kinematic parameters, the peak knee flexion and peak ankle dorsiflexion in early stance on the paretic side in patients with RHD were smaller than those in patients with LHD.
